# Neurofeedback and neuroplasticity of visual self-processing in depressed and healthy adolescents: A preliminary study

**DOI:** 10.1016/j.dcn.2019.100707

**Published:** 2019-09-11

**Authors:** Karina Quevedo, Guanmin Liu, Jia Yuan Teoh, Satrajit Ghosh, Thomas Zeffiro, Natasha Ahrweiler, Na Zhang, Riley Wedan, Sewon Oh, Guerson Guercio, Christian Paret

**Affiliations:** a Department of Psychiatry at the University of Minnesota (U of M), United States; b Center for Healthy Minds, University of Wisconsin-Madison, United States; c Department of Radiology, University of Maryland, United States; d Department of Otolaryngology, Harvard Medical School, McGovern Institute for Brain Research at the MIT, United States; e REACH Institute, Department of Psychology, Arizona State University, United States; f Department of Psychosomatic Medicine and Psychotherapy, Central Institute of Mental Health Mannheim, Heidelberg University, Germany

**Keywords:** ESOM, Emotion Self-Other Morph, ESOM_NF, Emotion Self-Other Morph Neurofeedback, ESOM_Pre, ESOM task presented before ESOM_NF task, ESOM_Post, ESOM task presented after ESOM_NF task, ROI, region of interest, fMRI, functional magnetic resonance imaging, BOLD, blood oxygen level dependent, Neurofeedback, Adolescence, Depression, Amygdala, Hippocampus, Dorsal anterior cingulate cortex, Self-face recognition

## Abstract

Adolescence is a neuroplastic period for self-processing and emotion regulation transformations, that if derailed, are linked to persistent depression. Neural mechanisms of adolescent self-processing and emotion regulation ought to be targeted via new treatments, given moderate effectiveness of current interventions. Thus, we implemented a novel neurofeedback protocol in adolescents to test the engagement of circuits sub-serving self-processing and emotion regulation.

**Methods:**

Depressed (n = 34) and healthy (n = 19) adolescents underwent neurofeedback training using a novel task. They saw their happy face as a cue to recall positive memories and increased displayed amygdala and hippocampus activity. The control condition was counting-backwards while viewing another happy face. A self vs. other face recognition task was administered before and after neurofeedback training.

**Results:**

Adolescents showed higher frontotemporal activity during neurofeedback and higher amygdala and hippocampus and hippocampi activity in time series and region of interest analyses respectively. Before neurofeedback there was higher saliency network engagement for self-face recognition, but that network engagement was lower after neurofeedback. Depressed youth exhibited higher fusiform, inferior parietal lobule and cuneus activity during neurofeedback, but controls appeared to increase amygdala and hippocampus activity faster compared to depressed adolescents.

**Conclusions:**

Neurofeedback recruited frontotemporal cortices that support social cognition and emotion regulation. Amygdala and hippocampus engagement via neurofeedback appears to change limbic-frontotemporal networks during self-face recognition. A placebo group or condition and contrasting amygdala and hippocampus, hippocampi or right amygdala versus frontal loci of neurofeedback, e.g. dorsal anterior cingulate cortex, with longer duration of neurofeedback training will elucidate dosage and loci of neurofeedback in adolescents.

## 1 Introduction

Depression upsurges in adolescence (Avenevoli et al., 2015) are linked to self-processing transformations (Chen et al., 1998). Self-processing ranges from quick self-recognition in group photographs to deliberate verbal self-appraisals. Self-development is a key maturational task of adolescence (Boyes and Chandler, 1992) enabled by developing midline cortical structures (Degeilh et al., 2015), a process that is linked to adolescent-typical heightened self-consciousness and susceptibility to peer influence (Sebastian et al., 2008). Therefore, adolescence is a crucial period to study circuits of self-processing via novel neuromodulatory procedures. Negative self-processing, ubiquitous in depression, is preferential perception and encoding of negative self-relevant information and neglect of positive self-relevant information (McCarty et al., 2007; Mezulis et al., 2004; Morris et al., 2008). Negative self-processing is a longitudinal risk factor of chronic depression and suicide attempts (Brunstein-Klomek et al., 2005; Brunstein Klomek et al., 2007; Cha et al., 2018; Morris et al., 2008; Orbach et al., 1998; Smith et al., 2006; Stange et al., 2015). Unfortunately, this core feature of depression resists change (Brunstein-Klomek et al., 2005; Brunstein Klomek et al., 2007; Morris et al., 2008; Orbach et al., 1998; Stange et al., 2015); perhaps due to circuit-level abnormalities that are not addressed by current treatments. Given that current interventions for adolescent depression underperform (Rush et al., 2006; Weisz et al., 2006), understanding how and whether depressed youth differ from healthy youth while heightening positive self-processing via neuromodulation, provides insights into depression’s pathophysiology and suggests how neuromodulation might lead to durable recovery. A promising procedure that might lead to new treatments is real-time functional magnetic resonance imaging neurofeedback, during which regional neural activity is visually displayed, enabling voluntary activity modulation from regions of interest “in vivo”. Neurofeedback combines neuromodulation and emotion regulation (Linhartova et al., 2019), thus it is well suited to non-invasively study dynamic self-processing and affect regulation in youth.

### 1.1 Face processing networks and depression

Unlike healthy emotional states, depression is characterized by exaggerated attention to and recall of negative versus positive information across stimuli (e.g. words, images, phrases (Platt et al., 2017) including social stimuli such as faces (Stuhrmann et al., 2013, 2011). The fusiform gyrus, amygdala, insula and hippocampus (Fusar-Poli et al., 2009) are part of the network enabling processing of emotional faces that distinguish depressed versus healthy individuals in literature reviews (Lai, 2014; Stuhrmann et al., 2011). Specifically, depressed patients evidence hyperactivation in the face processing network (amygdala, insula, parahippocampal gyrus, fusiform gyrus, and putamen) to negative faces and hypoactivation to positive faces (Lai, 2014; Stuhrmann et al., 2011); a bias that is present across development (Barlow et al., 2012; Beesdo et al., 2009; Chai et al., 2015; Leppanen et al., 2017; Monk et al., 2008; Nejad et al., 2013). Critically, these limbic and cortical abnormalities portend long term consequences. Slower identification of happy faces and faster of sad faces predicted onset of depression in adolescents over an 8-year period (Vrijen et al., 2016); demonstrating the need to target their underlying networks (e.g. amygdala and hippocampus and/or medial prefrontal cortex structures) early in life to forestall chronic lifelong depression.

To our knowledge, ours is the only research about self-face processing in depressed youth. Our work showed decreased activity in both frontotemporal [anterior cingulate cortex, posterior cingulate cortex, fusiform and medial prefrontal cortex] and limbic networks (bilateral hippocampus, amygdala and caudate) during self-face recognition, particularly during recognition of happy self vs. happy other faces (Alarcón et al., 2019; Quevedo et al., 2018, 2016). This suggests that hyporesponsive face-processing networks are exacerbated for the self-face in depressed youth. Critically, those neural abnormalities distinguished depressed youth with recent suicide attempts (Alarcón et al., 2019; Quevedo et al., 2016). Thus, hyporesponsive self-face processing networks might be a biomarker for depression and suicide risk that novel procedures such as neurofeedback ought to target. In summary, depression-specific biases toward negative or neutral faces (limbic hyperactivation) as well as biases away from positive faces (limbic hypoactivation) are present across development. These biases might be conspicuous for happy self-face processing in depressed youth; and right amygdala and frontotemporal circuits during self-face processing might be markers of severely depressed youth with suicide attempts (Alarcón et al., 2019; Quevedo et al., 2016). We sought to counter those biases by increasing frontotemporal and limbic activity during happy self-face processing via neurofeedback in depressed adolescents.

Face recognition (ours and others’) is enabled by the fusiform cortex and limbic structures such as the amygdala and hippocampus and self-recognition engages midline cortical structures, including the anterior cingulate cortex, posterior cingulate cortex, precuneus, inferior and superior temporal gyrus, and regions (Brodmann areas 10, 9) of the medial prefrontal cortex (Phan et al., 2004; Sugiura et al., 2005). Given that overlapping networks enable both self- and unfamiliar face recognition, we can use unfamiliar faces to control for a neurofeedback condition that paired self-face recognition and autobiographical memory recall. Thus, our control condition entailed a working memory task - which like self-processing elicits Brodmann area10 and anterior cingulate cortex activity (Woodward et al., 2006) - plus recognizing unfamiliar faces, which elicits fusiform and amygdala activity (Sabatinelli et al., 2011). To summarize, neglect of positive self-faces, ubiquitous negative self-processing in depression, added to the importance of adolescent self-development, prompted the selection of happy self-faces and happy unfamiliar faces as, respectively, the neurofeedback cue and control condition in the new protocol.

### 1.2 Neural function in depression and autobiographical memory

The amygdala and hippocampus complex enables our affective memories and this neural complex is reciprocally interconnected with the ventral and medial prefrontal cortex, posterior cingulate cortex, and precuneus to enable emotion regulation, self-processing and emotional autobiographical memory (Belzung et al., 2015; Doré et al., 2018). For example, amygdala-hippocampus-midline cortical structures connectivity strengthens as positive affect or arousal increases during autobiographical memory recall (de Voogd et al., 2017d; Nawa and Ando, 2019). Plentiful research shows abnormal amygdala and hippocampus activity in depression during self- and emotional processing (Belzung et al., 2015; Benson et al., 2014; Hastings et al., 2004; Tahmasian et al., 2013; Zheng et al., 2017). We found that depressed youth exhibited lower amygdala and hippocampus during recognition of happy self-faces versus recognition of happy other-faces compared to healthy youth (Quevedo et al., 2018). Suggesting that the neural substrates of emotional saliency and encoding of positive self-relevant information could be potential neural loci of neurofeedback training. Reviews have concluded that self-processing during depression is associated with abnormally heightened frontal midline cortical structures activity across modalities of self-processing (Li et al., 2017) and -pertinent to our choice of neural loci- with dysfunctional amygdala and hippocampus emotion regulation networks (Nejad et al., 2013). Given disrupted amygdala and hippocampus and midline cortical structure function in depression and their role in self-face processing (Phan et al., 2004; Sugiura et al., 2005); we tested amygdala and hippocampus as a loci of neurofeedback during adolescence: a formative period for self and emotion processing (Greenberg et al., 2004; Murphy et al., 2010). Given the protocol’s novelty and the absence of prior neurofeedback adolescent research, we were aware that youth could have engaged other areas than those reported in adult neurofeedback studies due to developmental differences in addition to methodological considerations. Thus, we sought to identify which loci - amygdala and hippocampus and/or loci within frontotemporal networks - were active in whole brain level analyses in adolescents. Additionally, by using face stimuli, we sought to elicit face-processing networks and test whether there would be different neuroplastic adaptations in depressed versus healthy youth during and after neurofeedback training while recognizing happy self vs. happy other-faces. Identifying such loci of neurofeedback could guide future placebo-controlled neurofeedback trials in adolescents.

### 1.3 Neurofeedback and neuroplasticity

Depressed and adults with borderline personality disorder who underwent amygdala neurofeedback, modulated that target’s activity and showed improvements in measures of self-processing (Linden et al., 2012; Paret et al., 2016; Young et al., 2017a, 2017b, 2018, 2014; Yuan et al., 2014; Zotev et al., 2011). In these populations, neurofeedback recruited medial prefrontal cortex-limbic networks including the dorsal anterior cingulate cortex (Zotev et al., 2011). Neurofeedback training also diminished anxiety and depression in active versus placebo-neurofeedback groups (Young et al., 2017b, 2018, 2014; Yuan et al., 2014) and corrected affect dysregulation and abnormal self-processing in behavioral tests (Young et al., 2017a). This suggests that neurofeedback’s effects in medial prefrontal cortex and amygdala circuits are a candidate mechanism of self-processing modification and a promising procedure to study neural plasticity. For example, left amygdala to medial prefrontal cortex functional connectivity during resting state increased after active neurofeedback compared to Placebo (Yuan et al., 2014), and in depressed adults’ amygdala activity during processing of happy faces was higher one week after active neurofeedback (Young et al., 2017a). Encouragingly, there was also increased saliency of positive stimuli and plasticity of the targeted areas after neurofeedback training. In fact, literature reviews concluded that engaging medial prefrontal cortex-limbic circuits during positive autobiographical memory recall (Young et al., 2018) and that circuits changes and symptoms’ improvement persisted for 2 months after neurofeedback training (Megumi et al., 2015; Rance et al., 2018; Young et al., 2014). In summary, neurofeedback appears to elicit beneficial neuroplasticity in networks that enable self-processing and emotion regulation, and such changes are associated with improved behavior in those domains (Young et al., 2017aa).

In this past research, depressed adults increased left amygdala activity by positive autobiographical memory recall cued to the word “Happy” (Young et al., 2017a, 2017b, 2018, 2014; Zotev et al., 2011). This was interpreted as learned control of neurophysiology during neurofeedback. Similarly, here we expected higher amygdala and hippocampus activity during neurofeedback cued to self-face recognition and lower activity during a count-backward plus other-face recognition condition as initial evidence of modulation of amygdala and hippocampus region of interest (ROI) activity. Additionally, post-training tasks have been used to test ensuing neuroplasticity (Young et al., 2017a, 2017b, 2018, 2014; Zotev et al., 2013). Given our interest in self-processing neurocircuitry, we used self-faces to cue positive autobiographical memory recall and neurofeedback training versus unfamiliar faces to cue a count-backwards condition. Accordingly, we tested training effects in self-other face recognition networks before versus after neurofeedback. Specifically, we sought to confirm whether training of amygdala and hippocampus activity induced neuroplasticity, and we examined associations between amygdala and hippocampus neurofeedback learning curves with amygdala and hippocampus mean activity during a self-other face recognition before and after training using the Emotion Self-Other Morph Query (ESOM-Q) task (Quevedo et al., 2018, 2016).

The current study determined the feasibility of real-time functional magnetic resonance imaging (fMRI) neurofeedback in depressed adolescents. We also aimed to gather data about the engagement of circuits sub-serving developmentally sensitive psychological distortions (i.e. negative self-processing and emotion dysregulation). Given blunted bilateral amygdala and hippocampus in depressed youth during happy self-face recognition (Quevedo et al., 2018), we tested whether neurofeedback could upregulate those regions’ activity. However, given non-existent neurofeedback research in this population, we sought to identify what key self-processing and emotion regulation loci –if not amygdala and hippocampus – could be targeted via neurofeedback. Answering this developmental question will guide future neurofeedback research in depressed adolescents. This was an early-phase study and, as noted in a review about control conditions or groups in neurofeedback designs (Sorger et al., 2019), it does not include a placebo group or condition. The aims were to: 1. implement a novel neurofeedback protocol in adolescents and identify if amygdala and hippocampus or other key loci within frontotemporal networks were up-regulated via neurofeedback, 2. determine if depressed youth differed from healthy adolescents during neurofeedback targeting cortico-limbic circuits of positive self-processing, and 3. test whether neurofeedback paired to the self-face versus a control condition of viewing a non-familiar face would change cortico-limbic networks underlying self-other face recognition.

Given the reviewed literature our hypotheses were as follows: first, youth would show higher amygdala and hippocampus and frontotemporal cortices activity (e.g. anterior cingulate cortex and medial prefrontal cortex) during feedback (happy self-faces plus voluntary positive autobiographical memory recall) versus control conditions (happy other-faces plus counting-backwards); second, there would be higher midline cortical structures or/and fusiform face-area engagement among depressed vs. healthy youth during neurofeedback; given well described hyperactivity in depressed individuals during self-processing (Lemogne et al., 2011, 2009; Lemogne et al., 2010; Nejad et al., 2013). Third we expected that amygdala and hippocampus modulation patterns during the task would differ between depressed and control youth, indexed by temporal changes in amygdala and hippocampus activity, referred to as neurofeedback learning slopes. We also expected that in region of interest (ROI) analyses, amygdala and hippocampus levels (i.e. slopes or overall mean activity) would be linked to amygdala and hippocampus activity during self-other face recognition before and after neurofeedback. However, we had no directional hypothesis given the novelty of the protocol. Fourth and final, we hypothesized that frontotemporal function underlying self-other face recognition – including midline cortical structures activity – would change after neurofeedback.

## 2 Methods

**Fig. 1:**
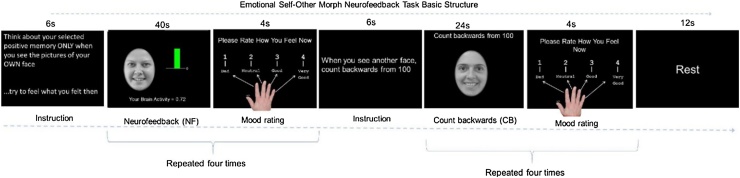
Emotion Self-Other Morph Neurofeedback (ESOM_NF): Conditions and timings.

**Fig. 2:**
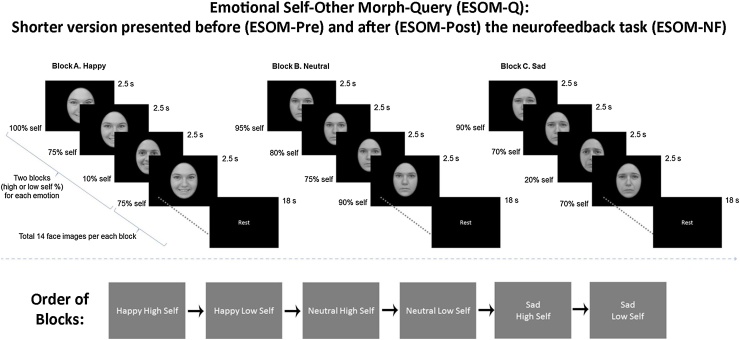
Emotion Self-Other Morph (ESOM): Conditions and timings presented before (ESOM_Pre) and after (ESOM_Post) the Emotional-Self-Other Morph Neurofeedback (ESOM_NF) task.

Participants were recruited from the community and inpatient units at the University of Minnesota. Exclusion criteria were general magnetic resonance imaging exclusions, psychosis, major medical or neurological disorders and substance use disorders. This study was approved by the University of Minnesota Institutional Review Board. Right-handed adolescents (N = 53, Table 1) were evaluated using both categorical Kiddie Schedule for Affective Disorders and Schizophrenia for School-age Children – Present and Lifetime version (K-SADS-PL) (Kaufman et al., 1997), and continuous Children’s Depression Rating Scale (CDRS) (Poznanski et al., 1984), clinical instruments and IQ was sampled with the Wechsler Abbreviated Scale of Intelligence (WASI) (Weschsler, 1999). A licensed clinical psychologist diagnosed presence or absence of depression. Pictures of the participants’ face with a happy, sad or neutral expression were taken as described in Quevedo et al. (2016). Depressed were on stable medication (Table 1). During a second session, and before scanning, participants wrote 5–6 positive autobiographical memories and identified peak positive moments with the experimenters. Participants completed a short version of the Emotion Self-Other Morph Query task (Fig. 2) before and after the Emotion Self-Other Morph Neurofeedback task (Fig. 1) and reported happiness and ease of recall during neurofeedback reported in supplemental text VI.

**Table 1:** Demographics and clinical presentation by diagnostic group.

	Healthy Controls	Depressed
	n = 19	n = 34
Suicide attempters*	n = 0_a_	n = 15_b_
***Age at Intake: M* (*SD*)**	16.26(1.19)	16.08(1.27)
***Age at Scanning: M* (*SD*)**	16.35(1.23)	16.11(1.25)
***IQ:M* (*SD*)**	115.32(9.12) _a_	108.35(10.84) _b_
**Sex**		
Male	7(36.84%)	10(29.41%)
Female	12(63.16%)	24(70.59%)
**Puberty: M(SD)**	4.53(0.65)	4.53 (0.68)
**Ethnicity**		
White	14(73.68%)	27(79.41%)
African American/Black	0	2(5.88%)
American Indian	0	2(5.88%)
Asian	3(15.79%)	0
Other Ethnicity	2(10.53%)	3(8.82%)
**Family Structure**		
Married	15(78.95%)	22(64.71%)
Living with partner	1(5.26%)	3(8.82%)
Separated-Divorced	3(15.79%)	5(14.71%)
Single	0	4(11.76%)
**Income**		
=>35K	0	6(17.65%)
35-75 K	7(36.84%)	9(26.47%)
+>75 K	12(63.16%)	19(55.88%)
Depression Severity (CDRS)*: M(SD)	19.21(3.56)_a_	49.85(16.14)_b_
Depression Diagnosis (K-SADS-PL)		
Major Depressive Disorder (MDD)	0	14
MDD with Psychotic Features	0	1
Dysthymia	0	4
Melancholic Depression	0	1
Depressive Disorder-NOS	0	15
Eating Disorders (K-SADS-PL)	0	2
Anxiety Disorders (K-SADS-PL)	0	22
PTSD (K-SADS-PL)	0	6
Disruptive Behavior Disorders (K-SADS-PL)	0	6
Substance Use Presence (K-SADS-PL)	0	2
**Medication**		
Antidepressants	0	26
Antipsychotics	0	2
Mood stabilizers	0	0
Anxiolytics	0	10

Note: Different a or b letter subscripts indicate significant statistical differences between the compared means. M=Mean; SD=Standard Deviation; NOS=Not otherwise specified.

* p < 0.05.

### 2.1 Tasks

#### 2.1.1 Emotion Self-Other Morph Neurofeedback (ESOM_NF)

This task (duration = 354 s) included a feedback condition comprised of four blocks (40 s). The first feedback block started with the participant seeing their own happy face (happy self-face) and were asked to increase amygdala and hippocampus activity displayed via a colored bar shifting up or down depending on values provided by MURFI software (Hinds et al., 2011), with green = activity > baseline and red = activity < baseline. To modulate amygdala and hippocampus activity, participants recalled happy autobiographical memories and were instructed both verbally before and during the task to elicit the positive feelings: “try to feel what you felt then”. Experimenters helped participants to recall the main topic of their key 5–6 memories before the start of the task. The control condition was comprised of 4 blocks (24 s) cued to an unfamiliar teen happy-face (happy other-face). Participants counted backwards from 100 with no feedback condition. Three rests occurred at onset (30 s), middle (onset = 80 s, duration = 12 s) and task’s end (onset = 342 s, duration = 12 s) comprising an implicit baseline condition. Participants saw instructions (6 s) after the first and second rests. After each feedback + self-face or count-backwards + other-face condition participants rated their affect (1=bad to 4=good). Feedback and count-backwards conditions alternated throughout (Fig. 1). Rest/baseline and counting-backwards conditions have served as contrasts to test neuromodulation’s effects in region of interest (ROI) during neurofeedback. Given the study’s preliminary nature we did not test a placebo condition. However, we chose a control condition entailing counting backward paired with recognizing an unfamiliar face. It was expected that this would engage areas supportive of both working memory (Woodward et al., 2006) and face processing (Sabatinelli et al., 2011) that overlap with those elicited by recalling positive autobiographical memories and self-face recognition during neurofeedback (Alarcón et al., 2019; Quevedo et al., 2018, 2016). Using this contrast condition also reflected our interest of testing how self vs. other-face recognition and their supporting neural networks would be affected by the use of those stimuli to prompt voluntary modulation (self-face recognition recognition) versus a different mental activity (other-face). The goal was to enhance positive self-processing by pairing voluntary positive autobiographical memory recall to happy self-faces and investigate how this would influence the circuitry of self vs. other face recognition.

#### 2.1.2 Emotion Self-Other Morph (ESOM)

A version of the Emotion Self-Other Morph Query task, duration = 360 s (Quevedo et al., 2018, 2016), was presented before (ESOM-Pre) and after (ESOM-Post) the neurofeedback task. Participants saw 112 faces with high or low percentages of self-features across three emotions (Happy, Neutral, or Sad) and indicated self versus other faces via button press. An initial rest block (duration = 30 ss) was followed by instructions (duration = 6 s). Blocks were presented in similar orders before and after Emotion Self-Other Morph Neurofeedback: Happy self-face, other-face, Neutral self-face, other-face, Sad self-face, other-face. Blocks lasted 35 s followed by a rest period (duration = 18 s). Before each emotion, instructions lasted 3 s. Within each self or other-face block, there were 2 instances of opposite condition to diminish response sets and maintain attention (e.g. self-face blocks included 2 other-faces). Stimuli, reaction time and accuracy were presented and collected using PsychoPy software (Peirce, 2007) (Fig. 2). This task was administered to test the effects of neurofeedback prompted by self-face and other-face stimuli upon the neurobiology of self-other face recognition.

### 2.2 Online analyses

MURFI software (Hinds et al., 2011) generated and sent amygdala and hippocampus activity values during the feedback condition displayed with PsychoPy (Peirce, 2007) during the Emotion Self-Other Morph Neurofeedback task using subject specific anatomical masks of the bilateral amygdala and hippocampus (See supplement text I). The bar representing amygdala and hippocampus values was updated as each new functional magnetic resonance imaging (fMRI) volume was acquired. Online subject head motion compensation was accomplished using the Siemens PACE/MoCo system (Thesen et al., 2000). Feedback automatically stopped if movement exceeded 4-3 mm repeatedly (which occurred in just one participant), but participants could re-initiate the Emotion Self-Other Morph Neurofeedback task. Region of interest (ROI) was localized anatomically during the multiband echo-planar imaging (EPI) series (target functional reference acquisition, see supplements) for each individual and mapped to individual’s T1 structural brain data. Data were collected using a 3.0 T Siemens Prisma MRI scanner with the 32 Channel receive only head coil. Structural 3D axial MPRAGE images were acquired for each participant (TR/TE: 2100 ms/3.65 ms; TI: 1100; Flip Angle 7°; Field of View: 256 × 256 mm; Slice-Thickness: 1 mm; Matrix: 256 × 256; 224 continuous slices), GRAPPA 2. Mean blood oxygen level dependent (BOLD) images were then acquired with a slice-accelerated gradient echo-planar imaging sequence during 6.08 min for the Pre- and Post- Emotion Self-Other Morph tasks and 6.02 min for the Emotion Self-Other Morph Neurofeedback task (2.4 mm^3^ voxels, covering 60 oblique axial slices; TR/TE = 1510/32.4 ms; FOV = 216 × 216 mm; matrix 90 × 90; Flip Angle 65°; multi-band acceleration factor 3).

### 2.3 Off-line analyses

SPM12 was used for all functional magnetic resonance imaging (fMRI) preprocessing and statistical analyses. Echo-planar imaging time series’ preprocessing included: (1) rigid body realignment for head motion correction, (2) slice timing correction, (3) rigid body co-registration of EPI with high resolution anatomical data, (4) spatial normalization to the Montreal Neurological Institute (MNI) anatomical space using unified segmentation, and (5) spatial smoothing (6 mm full width at half maximum). Head motion outliers in echo-planar imaging time series were identified and corrected using the Artifact Detection Tools with a scan-to-scan movement threshold of 0.5 mm and a scan-to-scan global signal change of 3 SD (www.nitrc.org/projects/artifact_detect/). For each subject, blood oxygen level dependent (BOLD) -contrast signal variance was modeled with a set of regressors using a general linear model. The total signal variance was decomposed into a task component, with inter-trial intervals as implicit baselines. Each task regressor was constructed by generating condition duration vectors and then convolving them with a canonical hemodynamic response function, allowing parameter estimates proportional to task-related neural activity per second. The full model for each subject comprised: (1) the condition regressors, (2) regressors modeling movement-related signal modulation, (3) outlier time points, (4) the mean signal for the session, (5) a discrete cosine transform basis set that modeled the low frequency, presumably artifactual, signal modulations at frequencies lower than 0.008 Hz and (6) realignment and censoring regressors for nuisance physiological noise. Parameter estimates were calculated using restricted maximum likelihood algorithm.

To examine Emotion Self-Other Morph Neurofeedback task conditions’ effects, a voxel-wise analysis of the feedback training periods used first-level feedback minus count-backwards contrasts in a one-sample *t*-test (critical voxel-level threshold: *p_FWE_ <* 0.05) including diagnosis, IQ and gender as predictors to identify regional effects.

Analyses of training effects in self vs. other face recognition networks were performed by calculating group differences before versus after the Emotion Self-Other Morph Neurofeedback task for the self vs. other face recognition task (ESOM_Pre vs. ESOM_Post) using paired *t*-tests on first-level contrast images representing the difference between ‘self’ and ‘other’ task conditions with IQ, gender, reaction time and accuracy as covariates (cluster forming threshold: *p_uncorr_*<0.001, and a critical cluster-level threshold: *p_FWE_ <* 0.05) for better resolution of structures, given large cortical networks engaged.

To test whether common regions were activated during both feedback vs count-backwards for Emotion Self-Other Morph (ESOM) Neurofeedback task and for ESOM_Pre versus ESOM_Post during self vs. other-face recognition; a conjunction analysis testing for the conjunction null (Friston et al., 2005) was conducted. Maps from both contrasts were at a cluster forming threshold of *p_uncorr_* <0.001 and multiplied. Clusters surviving a threshold of *p_FWE_<*0.05 were reported.

A general linear model (GLM) with Emotion Self-Other Morph Neurofeedback conditions (feedback, count-backwards) as within subject factors covarying for IQ and gender (Table 1) was used to test diagnostic group effects using *p_uncorr_*<0.001. A combined voxel-height and cluster-extent threshold was calculated to control for Type 1 error with Monte Carlo simulations in AFNI (v. 18.2.06) (Cox, 1996) and 3dClustSim, α = 0.01. Smoothness estimates entered in 3dClustSim (11.80 11.47 12.42) were calculated by 3dFWHMx. Only clusters > = 143 voxels were significant.

Accuracy and reaction time of self-other face recognition during pre- and post-Emotion Self-Other Morph and affect ratings and reaction times during Emotion Self-Other Morph Neurofeedback, were examined with repeated measure analysis of variance (ANOVA) with group as between subject condition covarying for IQ and gender.

### 2.4 Amygdala and hippocampus activity analysis

Given that region of interest (ROI) analyses are common in neurofeedback studies (Cohen Kadosh et al., 2016; Linden et al., 2012; Ruiz et al., 2013; Young et al., 2017a, 2017b, 2018, 2014; Zotev et al., 2013) and because we hypothesized higher ROI activation for feedback + self-face versus count-backwards + other-face conditions, each subject’s first level activity maps and subject-specific amygdala and hippocampus masks employed during the Emotion Self-Other Morph Neurofeedback task, were used to derive a mean within subject blood oxygen level dependent (BOLD)-contrast activity value for the 8 blocks of Emotion Self-Other Morph Neurofeedback and the 6 blocks of ESOM-Pre and ESOM-Post. These subject specific ROI values were used to test associations of amygdala and hippocampus activity before vs. after Emotion Self-Other Morph Neurofeedback to amygdala and hippocampus activity during Emotion Self-Other Morph Neurofeedback as well as group differences during Emotion Self-Other Morph Neurofeedback using linear mixed models in SPSS 25 (Supplemental text II, Fig. S.2). ROI analyses used individual mean signals after pre-processing and movement correction for feedback and count-backwards conditions versus baseline.

Individual intercepts (β0i) were modeled as random effects and mean intercept (γ00), slopes (linear, quadratic, cubic slopes = γ01, γ02, γ03) and predictors of interest as fixed effects. Linear change over time and inflection points during Emotion Self-Other Morph Neurofeedback were modeled with a linear (γ01), quadratic (γ02) or cubic predictors (γ03). Limited degrees of freedom prevented testing inflection points beyond cubic. An identity covariance structure for the random effects fitted best both tasks’ time series. For Emotion Self-Other Morph Neurofeedback, we tested 22 predictors. To select final models, predictors were removed one at a time starting with the least significant. Nested models were compared via a χ^2^ -2LL fit difference (Singer and Willett, 2003). Final models include only significant predictors, including interactions, but can exclude main effects if simpler models better fitted the data (Singer and Willett, 2003). Simple slopes tests, keeping all other predictors at mean values, confirmed direction of interactions (Table 2).

**Table 2:** Predictors of Amygdala and Hippocampus Activity Time Series during the Emotion Self-Other Morph Neurofeedback Task.

Effect	Estimate ϒ	SE ϒ	*Df*	T	P
Intercept	0.01	0.03	151	4.28	<0.01
Linear Slope	−0.11	0.03	371	−4.26	<0.01
Effect of Emotion Self-Other Morph Neurofeedback Condition					
Feedback+Happy self-face	0.03	0.02	371	2.26	*<0.05*
Count-backwards+Happy other-face a					
Quadratic: 1 st Learning Slope (**γ02 _Emotion Self-Other Morph Neurofeedback_**)	0.32	0.01	371	3.45	<0.01
Cubic: 2^nd^ Learning Slope (**γ03 _Emotion Self-Other Morph Neurofeedback_**)	−0.003	0.001	371	−3.07	<0.01
Group * 1^st^ Learning Slope (**γ02 _Emotion Self-Other Morph Neurofeedback_**)					
Controls	0.01	0.001	339	2.03	*<0.05*
Depressed a					
Effect of Gender					
Females Males a	−0.05	0.02	52	−2.09	*<0.05*
Effect of Amygdala and Hippocampus Activity during ESOM_Post Other Face minus ESOM_Pre Other Face	0.04	0.02	53	0.45	*<0.05*

a = reference group or condition.

To further confirm whether the amygdala and hippocampus activation was significant in the contrast of feedback versus count-backward or feedback versus baseline, we conducted ROI analyses with SPM12. Specifically, contrasts of feedback + self-face versus count-backward + other-face and feedback + self-face versus baseline were created for each participant. One-sample t-tests (including diagnosis, IQ and gender as predictors) were conducted on the group level (cluster forming threshold: *p_uncorr_*<0.001, and a small-volume corrected threshold: *p_FWE_ <* 0.05) for the bilateral amygdala and hippocampus ROI.

## 3 Results

### 3.1 Emotion Self-Other Morph Neurofeedback amygdala and hippocampus ROI activity

**Fig. 3:**
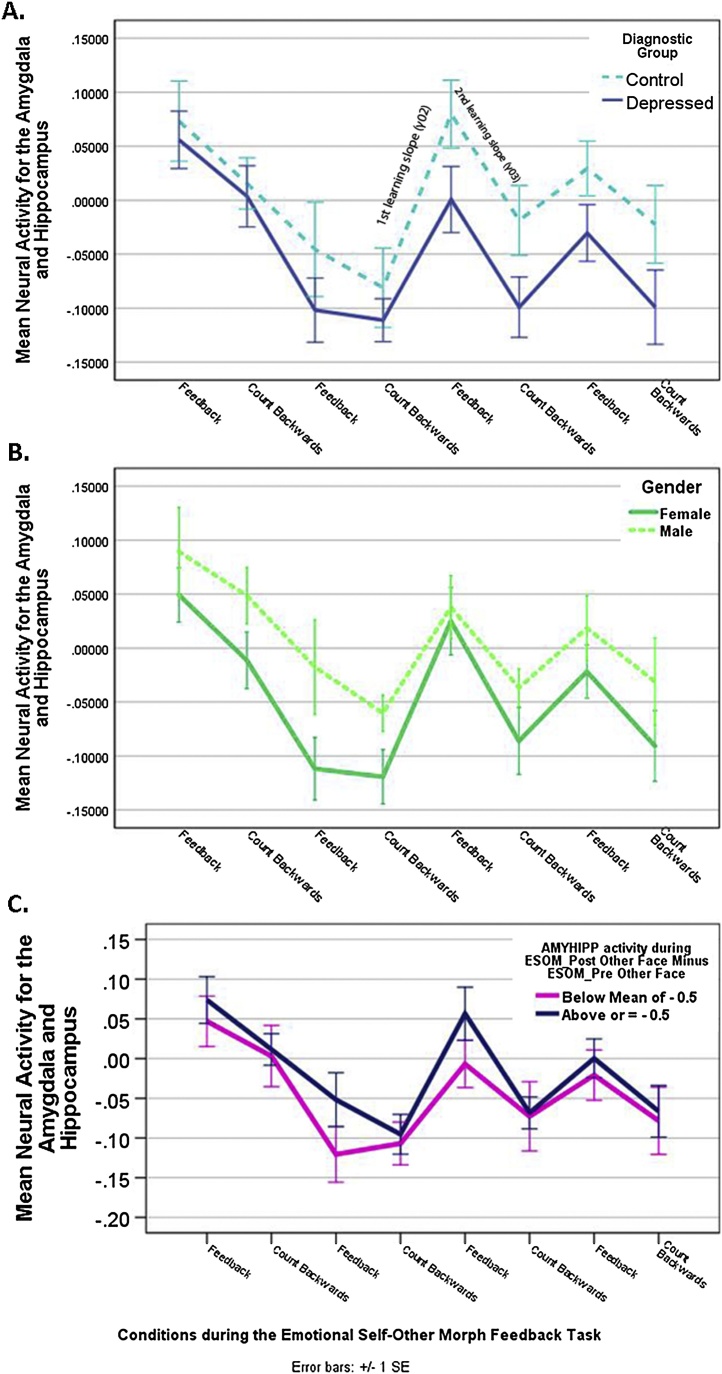
Amygdala and hippocampus activity during a neurofeedback task (ESOM_NF) that targeted self-processing was predicted by diagnostic group (Panel A), Gender (Panel B), and it was associated with changes in amygdala and hippocampus activity levels (Panel C) during recognition of other faces after versus before the neurofeedback task (ESOM-Post vs. ESOM-Pre). Finally, all youth showed higher mean amygdala and hippocampus activity during the feedback vs. the count-backwards condition.

**Fig. 4:**
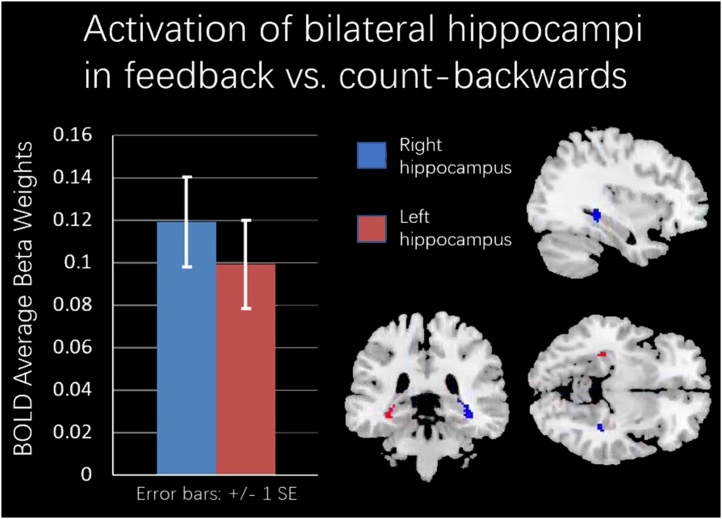
The bilateral hippocampus activity was significantly active in ROI analyses for feedback versus count-backwards conditions. However, the amygdala did not show significant activity.

Table 2 shows a main effect of neurofeedback condition in linear mixed model analyses, such that participants showed higher amygdala and hippocampus activity during the feedback versus the count-backwards condition (Fig. 3. Supplemental Fig. S.2) even while controlling for multiple significant covariates, M_Count-backwards_= −0.34, SE = 0.1, M_Feedback_ = 0.01, SE = 0.1. However, youth showed decreased amygdala and hippocampus activity over time (negative linear slope). A positive quadratic slope indicated that (after an initial activity drop) youth began increasing and decreasing amygdala and hippocampus activity concomitantly with feedback or count-backwards condition (Fig. 3). An increased 1^st^ neurofeedback learning slope (**γ02_Emotion Self-Other Morph Neurofeedback_**, Fig. 3) was followed by a drop during the 3^rd^ count-backwards condition, given a significant negative cubic predictor (2^nd^ neurofeedback learning slope = **γ03_Emotion Self-Other Morph Neurofeedback_**). Those inflection points timed to feedback and count-backwards conditions, suggest learning effects (Fig. 3). A group by 1^st^ learning slope (**γ02_Emotion Self-Other Morph Neurofeedback_**) interaction shows that controls have steeper slopes compared to depressed (Fig. 3, Panel A). Simple quadratic slopes contrasts confirmed this finding (**ϒ02_Emotion Self-Other Morph Neurofeedback_**_Controls minus Depressed_) = 0.001; SE = 0.001; *F*(1,339) = 4.13; p < 0.05. Males showed higher amygdala and hippocampus activity compared to females (Fig. 3, Panel B). Furthermore, we found that higher amygdala and hippocampus activity during neurofeedback was associated to higher amygdala and hippocampus activity for other-faces recognition after vs. before neurofeedback (other-face post minus other-face pre amyhipp activity) (Fig. 3, Panel C). Results from one-sample t-tests region of interest (ROI) analyses showed that bilateral hippocampi (Fig. 4, Table 3) were significantly activated in the contrast of feedback + self-face versus count-backward + other-face (right hippocampus: [34 −34 −10], cluster size (k) = 72; left hippocampus: [−36 −26 −14], k = 49). No significant results were found for the contrast of feedback versus baseline.

**Table 3:** Activity during neurofeedback versus count-backwards conditions during Emotion Self-Other Morph Neurofeedback.

### 3.2 Whole brain level effects of Emotion Self-Other Morph Neurofeedback

**Fig. 5:**
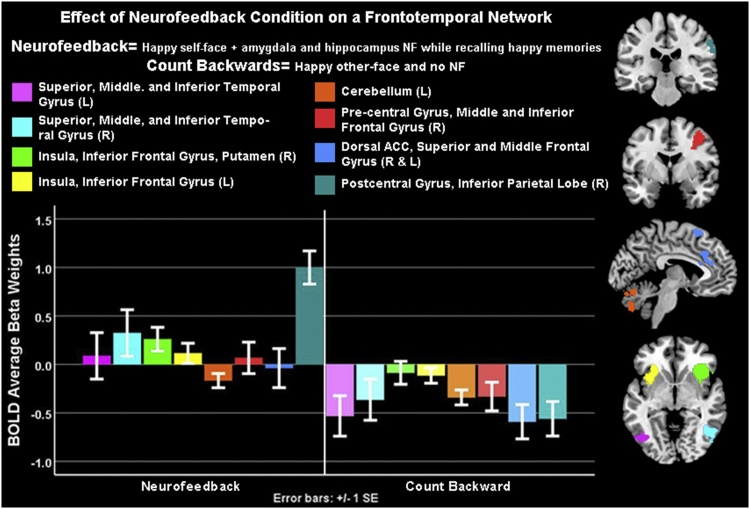
Emotional-Self-Other Morph Neurofeedback (ESOM_NF) Task Effects: All participants showed higher activity during the neurofeedback (NF) condition cued to happy self-face images versus the count-backwards cued to happy other-faces image conditions in frontotemporal cortices, e.g. anterior cingulate cortex, and sensorimotor areas in whole brain level analyses (Table 3). For whole brain activity intensity maps please see supplemental information.

One-sample *t*-tests showed that neural activity was higher during the feedback vs. count-backwards condition contrast in: bilateral inferior frontal gyri; anterior insula; putamen; bilateral superior, middle and inferior temporal gyri; left intermediate and lateral cerebellum; right middle and inferior frontal gyri; bilateral superior middle frontal gyri and dorsal anterior cingulate cortex (Fig. 5, Table 3). There were no significant areas of activity linked to IQ or gender.

### 3.3 Group by Emotion Self-Other Morph Neurofeedback conditions

**Fig. 6:**
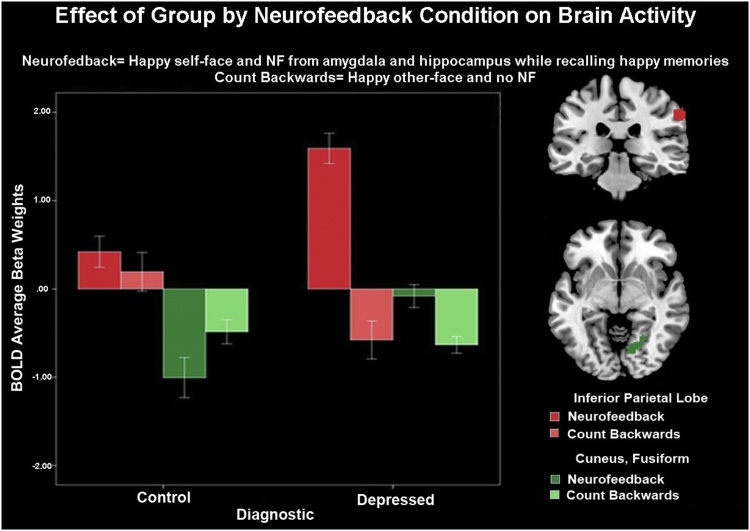
Group by neurofeedback condition: During the Emotional-Self-Other Morph Neurofeedback task, depressed adolescents showed higher activity in the inferior parietal lobe, cuneus and fusiform during the neurofeedback versus the count-backwards condition. Healthy control showed no activity differences in those areas due to condition.

General linear model analyses showed that depressed showed higher activity during feedback vs. count-backwards conditions in right inferior parietal lobe, cuneus and fusiform cortices while controls showed similar activity in those areas for both conditions (Fig. 6, Table 3).

### 3.4 Brain activity before (ESOM_Pre) and after (ESOM_Post) Emotion Self-Other Morph Neurofeedback

**Fig. 7:**
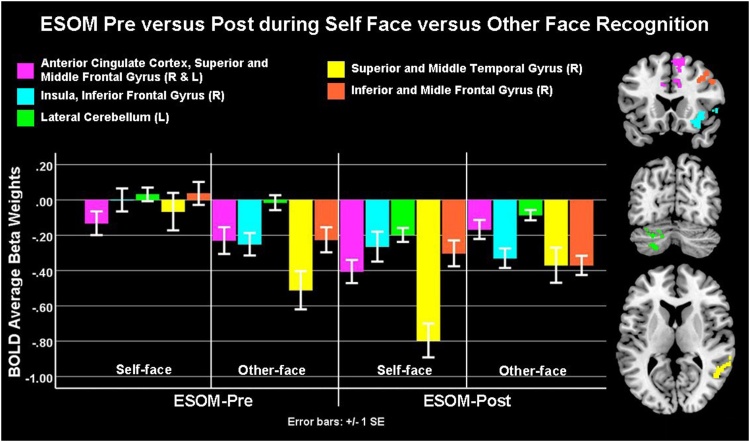
Emotional Self-Other Morph (ESOM-Pre) versus ESOM-Post Activity Changes: Before the neurofeedback training task (i.e. ESOM_NF) during a self- versus other face recognition task (i.e. Emotional Self Other Morph) all participants showed higher neural activity during self- vs. other face recognition. After neurofeedback training during ESOM-Post all participants showed lower neural activity during self- versus other-face recognition. For whole brain activity intensity maps please see supplements Fig. 7 online.

Paired *t*-tests showed that before neurofeedback, youth showed higher activity during self-face recognition in the anterior cingulate cortex, superior, mid-frontal and temporal gyri and insula compared to other-face recognition. However, after neurofeedback they showed higher activity during other-face in those areas compared to self-face recognition (Fig. 7, Table 4). Critically, there were no differences in those areas’ activity for other-face recognition after vs. before neurofeedback. The difference was due to *lower brain activity during self-face recognition after vs. before neurofeedback*. Finally, there were no differences between groups or due to IQ, gender, accuracy or reaction time.

**Table 4:** Activity for Emotion Self-Other Morph_Post versus Pre during Self Face versus Other Face recognition.

Whole-Brain Results	Voxels	Hemisphere	MNI Coordinates			T
			x	Y	Z	
Anterior Cingulate Cortex, Superior and Middle Frontal Gyrus, Brodmann Areas 6, 8, 9, 10, 24, 32	1774	Right and Left	02	46	22	5.55
Insula, Inferior Frontal Gyrus, Brodmann Areas 13, 45, 47	447	Right	36	22	−06	4.41
Lateral Cerebellum	374	Left	−26	−72	−42	4.37
Inferior and Middle Frontal Gyrus, Brodmann Area 9	230	Right	34	06	30	4.17
Superior and Middle Temporal Gyrus, Brodmann Areas 21, 22, 39	197	Right	64	−42	04	4.02

### 3.5 Common activity in Emotion Self-Other Morph Neurofeedback and ESOM_pre vs ESOM_post

As shown in Fig. S.6 in the supplements, and Table 5 conjunction analyses yielded that the anterior and middle cingulate extending to dorsomedial prefrontal cortex and the right insula extending to lateral orbitofrontal cortex, inferior frontal gyrus, and the cerebellum were commonly activated in the contrasts of both feedback + self-face vs. count-backwards + other-face during the neurofeedback task and self vs. other-face recognition. Suggesting that this might be an important area to target during neurofeedback training.

**Table 5:** Common Activity during Emotion Self-Other Morph (ESOM) Neurofeedback (feedback vs count-backwards) with ESOM-Post versus Pre (Self versus Other Face) recognition.

Whole-Brain Results	Voxels	Hemisphere	MNI Coordinates		
			x	Y	Z
Anterior Cingulate Cortex, Dorsomedial Prefrontal Cortex, Middle Cingulate Cortex, Brodmann Areas 9, 24, 32	1460	Left and Right	0	26	32
Insula, Lateral Orbitofrontal Cortex, Inferior Frontal Gyrus, Brodmann Areas 45, 47	298	Right	36	24	−6
Lateral Cerebellum	201	Left	−16	−72	−32

### 3.6 Affect ratings, accuracy, and reaction time

**Fig. 8:**
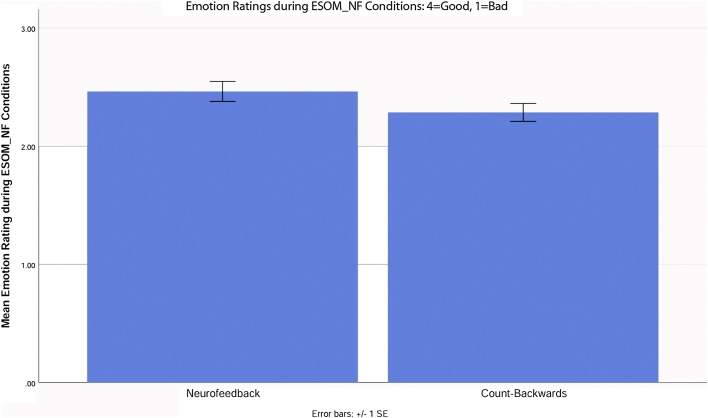
All participants, regardless of diagnostic group, reported higher positive feelings after neurofeedback versus after count-backwards blocks during the Emotion Self-Other Morph Neurofeedback (ESOM_NF) task.

General linear model analyses showed that during Emotion Self-Other Morph (ESOM) Neurofeedback all youth rated their affect as more positive after feedback + self-face + autobiographical memory versus count-backward + other-face blocks (i.e. feedback vs. count-backwards conditions), *F_Emotion Self-Other Morph Neurofeedback Condition_* (1, 21) = 6.6, p < 0.05 (Fig. 8). During ESOM-Post vs. Pre all youth had higher accuracy, *F_Time_*(1, 51) = 4.42, p < 0.05, 3), and a time by face interaction indicated higher accuracy for other-face recognition before neurofeedback yet higher accuracy for self-face recognition after neurofeedback, F_Self_ vs_.Other*Time_(1, 51) = 9.1, p < 0.05 in all participants. During Emotion Self-Other Morph Neurofeedback all youth were slower to rate their affect after feedback vs. count-backwards blocks, *F_Emotion Self-Other Morph Neurofeedback Condition_* (1, 21) = 6.6, p < 0.05. During ESOM all youth were slower to recognize happy versus neutral and sad faces *F_Emotion_*(2, 82) = 7.580, p < 0.01. A group by time interaction indicated that controls recognize self-faces faster during ESOM-Pre but recognized other-faces faster during ESOM-Post, yet depressed recognized other-faces faster during ESOM-Pre but recognized self- and other-faces equally fast during ESOM-Post, F_Time*Self_vs._Other*Group_(1, 41) = 4.903, p < 0.05 after neurofeedback.

## 4 Discussion

To our knowledge, there are only two neurofeedback studies in children and adolescent samples (Alegria et al., 2017; Cohen Kadosh et al., 2016). This is the first study showing neurofeedback’s feasibility in depressed adolescents and our results could be critical to guide future fast-track clinical trials. Our present and past data suggest that longer duration of amygdala and hippocampus neurofeedback could be needed to elicit whole brain level activity in these limbic loci and that neurofeedback ought to target the dorsal anterior cingulate cortex versus limbic sites such as the right amygdala (Alarcón et al., 2019) or the bilateral hippocampus (significant in ROI analyses) in depressed adolescents. These loci are critical for affect-regulation and self-processing (dorsal anterior cingulate cortex), implicit emotional experiences (right amygdala) and emotional memories (hippocampus). Clinical effectiveness of targeting the dorsal anterior cingulate cortex versus limbic sites in adolescent neurofeedback studies are important because the former is active in whole brain level analyses and after neurofeedback for self vs. other face recognition, yet the hippocampi were significant in region of interest (ROI) analysis and the amygdala and hippocampus showed signs of neuromodulation in linear mixed model analysis.

### 4.1 A novel neurofeedback protocol effects in depressed and healthy adolescents

As hypothesized, youth showed higher amygdala and hippocampus activity during feedback (happy self-face plus positive autobiographical memory recall) vs. count-backwards (happy other-face) conditions in ROI analyses (Table 2: Main effect of Emotion Self-Other Morph Neurofeedback Condition and Fig. 3, Fig. 4). High amygdala and hippocampus activity differences during feedback were present even after controlling for significant effects of gender, group by slope interactions, and amygdala and hippocampus activity after vs. before neurofeedback during self vs. other face recognition (Table 2). Linear mixed models sequentially eliminate non-significant predictors until the best model fit is achieved, thus it is a strong possibility that youth were able to voluntarily modulate amygdala and hippocampus activity during feedback vs. count-backwards. In the absence of a placebo condition and of whole brain level effects in the targeted loci, this remains a tentative interpretation. Nonetheless, significant hippocampi activity in small volume corrected ROI analyses (Fig. 4) during feedback vs. count-backward conditions, indicates higher activity in the targeted loci while recalling positive memories and receiving neurofeedback. Not significant amygdalae activity suggest that happy self and other faces might have elicited similar amygdalae engagement during, respectively feedback and count-backwards, and/or that a higher dosage is needed to engage the amygdalae.

Methodological factors could explain lack of amygdala and hippocampus engagement in whole brain level analyses. First, prior neurofeedback trials (left amygdala ROI) that yielded whole brain level activity, lasted ∼ 22 min across 4 runs (Young et al., 2014). Our task lasted 5.54 min. Given that all youth reported higher positive affect during feedback vs. count-backward blocks (Fig. 8), this protocol might induce beneficial plasticity in frontotemporal-limbic networks in depressed youth using longer Emotion Self-Other Morph Neurofeedback duration like those used in adult trials. In addition to differences in dosage, our results suggest alternative loci of neurofeedback. For example, some experts in neurofeedback advocate for targeting smaller locus, and our selected region of interest (ROI) was larger than ROI’s used in prior studies (Young et al., 2017a, 2017b, 2018, 2014; Zotev et al., 2013). Perhaps just a right amygdala target (a potential marker of suicide attempts in youth (Alarcón et al., 2019) or a bilateral hippocampus target (a potential marker of adolescent depression (Quevedo et al., 2018) would evidence modulation in linear mixed models and activity at the whole brain level. Given, the tasks’ novelty and the targeted adolescent population, our results inform future adolescent neurofeedback trials and use of stimuli of unique saliency for adolescent neurofeedback during this sensitive developmental period.

In whole brain level analyses, youth engaged frontotemporal (including the dorsal anterior cingulate cortex) and somatosensory cortices during feedback (happy self-faces plus voluntary positive autobiographical memory recall) vs. the control condition (happy other-faces plus counting-backwards). The reported main effects of Emotion Self-Other Morph Neurofeedback task conditions are like those yielded by adult studies (Young et al., 2017a, 2017b, 2018, 2014; Zotev et al., 2013). Specifically, the neurofeedback protocol recruited neural networks that support emotion regulation and autobiographical memory recall, interoceptive awareness, imitation, and self- and social processing (Okon-Singer et al., 2015; Sebastian et al., 2008; Sliz and Hayley, 2012). These areas have reciprocal anatomical and functional connections with the amygdala (Kim et al., 2011; McCormick et al., 2015; Svoboda et al., 2006) and hippocampus (Campbell et al., 2018; Geng et al., 2016; Gluth et al., 2015). As stated, future research ought to test right amygdala and hippocampus versus dorsal anterior cingulate cortex as competing loci of neurofeedback in adolescents with longer protocols.

Altered frontotemporal (particularly anterior cingulate cortex) and lower limbic activity during processing of positive vs. neutral or negative faces characterizes depression in adults (Barlow et al., 2012; Lai, 2014; Nejad et al., 2013; Stuhrmann et al., 2011) and self vs. other face recognition in adolescents (Alarcón et al., 2019; Quevedo et al., 2018, 2016). It is thus encouraging that depressed youth increased frontotemporal and amygdala and hippocampus ROI activity during the neurofeedback condition similarly to healthy youth while recalling positive autobiographical memories. Reported higher positive affect and slower reaction time in affect ratings after feedback versus count-backwards blocks in both groups, suggest that the neurofeedback procedure elicited higher positive affect. All youth might have been more thoughtful while rating their affect after feedback blocks given their significantly slower reaction times. Lack of group by rating interactions suggest that feedback blocks increased positive affect and/or positive self-processing in all youth.

### 4.2 Diagnostic group differences and similarities

We hypothesized different patterns of whole brain level or ROI activity between diagnostic groups. As reported, both groups showed similar average learning slopes, with increased amygdala and hippocampus during feedback vs. count-backwards condition in ROI analyses (Fig. 3. Table 2) and similar engagement of frontotemporal cortices during feedback vs. count-backwards (Fig. 5, Table 3). However, there were indications of faster learning among controls in ROI analyses. Specifically, a steeper 1^st^ learning slope compared to depressed youth in amygdala and hippocampus activity levels (Fig. 3, Panel A). A tentative explanation is that, after an initial struggle during the first three blocks, controls might upregulate amygdala and hippocampus via recall of positive memories more deftly compared to depressed adolescents. However, both groups appear to become similar in mean regulatory patterns for following blocks. Finally, later inflections points (beyond cubic and quadratic polynomials) could not be modeled, due to instability of beta weights for higher order polynomials. Nevertheless, Fig. 3 suggests similar learning effects as amygdala and hippocampus activity increases during feedback and decreases during the count-backward condition.

The initial drop in amygdala and hippocampus during the neurofeedback blocks (Fig. 3) is interesting. Amygdala and hippocampus signal decrease (and recovery by the 3^rd^ neurofeedback block) might reflect saliency-driven neural activity in response to a novel stimulus (self-face plus a bar of changing color). This novelty effect might be followed by habituation in later blocks. Additionally, after initial saliency-driven high activity, youth might struggle to regulate the signal, yet by the 3^rd^ neurofeedback block they might be mastering the task. Upon observing amygdala and hippocampus time series for the Emotion Self-Other Morph task before and after neurofeedback, the same pattern of high activity followed by a significant linear decline was found (Supplemental Fig. S.5). This suggests that saliency-habituation driven effects were not unique to the Emotion Self-Other Morph Neurofeedback task, but that instead initial high amygdala and hippocampus activity is present at the onset of every task administered. Perhaps a combination of initial high saliency, habituation and learning might explain the findings. Our explanation is bolstered by known habituation effects of the human amygdala during visual processing of facial expressions (Breiter et al., 1996). Faces were used as cues for neurofeedback and count-backwards blocks and during the task administered after and before neurofeedback training, thus a ROI comprised by the amygdala and hippocampus might habituate initially and youth were able to up-regulate the ROI after habituation effects stabilized. This needs to be tested with a longer protocol to confirm if three ∼ 40 s neurofeedback blocks are indeed a habituation period in youth. Additionally, counterbalancing orders of neurofeedback and count-backwards would test if high activity occurs regardless of stimuli. Minimally, our results suggest that a practice neurofeedback task is needed in future designs. If true, this would increase the number of blocks for which neuromodulation (i.e. higher activity during feedback versus a control condition) is evident during the actual Emotion Self-Other Morph Neurofeedback runs.

Regarding whole brain activity analyses, as we hypothesized, depressed showed higher activity during recall of positive autobiographical memories plus feedback cued to happy self-faces compared to healthy adolescents. Specifically, depressed youth engaged areas associated with face processing (fusiform) (Eifuku, 2017; Kanwisher and Yovel, 2006), visual association (cuneus) (Wichmann and Muller-Forell, 2004) and self-social processing (inferior parietal lobe) (Igelstrom and Graziano, 2017) more than healthy youth during feedback vs. count-backwards condition.

The groups reported similar ease of autobiographical memory recall, yet, results suggest greater cognitive effort while recalling memories and/or eliciting positive emotions as instructed by the task. This could be due to heightened response to self-face images and/or to engagement of more intense social cognition and/or visual associations during positive autobiographical memory recall among depressed youth. These results may be a marker of depression’s pathophysiology. Alternatively, these is how neurofeedback cued to visual positive self-processing might exert its influence in depressed youth, given that all youth report higher positive affect after the neurofeedback condition. Only follow-up research with additional conditions (e.g. happy self-faces plus autobiographical memory recall and no feedback) or a placebo condition or group could clarify these results. Finally, all except 5 depressed youth were medicated, thus medication effects could not be tested in this research. While medication was unrelated to both whole brain and region of interest brain activity during this protocol, its effects were noted with the original Emotion Self-Other Morph Query task (Quevedo et al., 2018).

### 4.3 Neurofeedback and networks of self-face recognition

We conjectured frontotemporal and limbic changes during self-other face recognition after neurofeedback as well as an association between amygdala and hippocampus activity levels across the Emotion Self-Other Morph (ESOM) Neurofeedback and the ESOM-Pre-Post tasks. We found higher frontotemporal cortical activity for self-face recognition before neurofeedback, but lower after neurofeedback (Fig. 7). Areas (anterior cingulate cortex and insula) that are part of the saliency network (Menon and Uddin, 2010) evidenced decreased activity during self-face recognition after neurofeedback via positive autobiographical memory recall paired to self-faces. Additionally, mean amygdala and hippocampus activity during the Emotion Self-Other Morph Neurofeedback task was linked to amygdala and hippocampus activity during other-face recognition after versus before neurofeedback in all youth. Both these results suggest similar effects of neurofeedback in corticolimbic areas regardless of diagnosis.

The Emotion Self-Other Morph (ESOM) Neurofeedback task might have induced neuroplasticity that affected the saliency of self-relevant stimuli given that neural changes were specific to the self-face condition during the ESOM-Post task. However, a placebo group and/or additional conditions (e.g. self-face plus positive autobiographical memory recall and no feedback condition) will better ascertain the specific process via which neuroplasticity took place. This would establish whether lower anterior cingulate cortex, insula, and cerebellum during self-face recognition after Emotion Self-Other Morph Neurofeedback was due to neurofeedback, self-face habituation, positive autobiographical memory recall or a combination of stimuli and mental activities. Minimally, analogous frontal (anterior cingulate cortex) and temporal cortical structures (right insula) and cerebellar heightened activity during feedback paired to the self-face subsequently decreased for self-face recognition after neurofeedback (Table 3, Table 4, Table 5). Of note, participants were more accurate for self vs. other-face recognition after versus before neurofeedback, this fact added to lower saliency network engagement for self-face after neurofeedback, might mean that self-recognition was facilitated, requiring less cognitive-affective coordination by the anterior cingulate cortex and the insula.

## 5 Limitations

This is the first neurofeedback protocol including depressed adolescents. The sample size (N = 53) was larger than the prior only two published neurofeedback studies of children (Alegria et al., 2017; Cohen Kadosh et al., 2016). However, a limitation of this study is the absence of significant amygdala and hippocampus activity in whole brain level analyses. Future research ought to increase the dosage (i.e. duration of neurofeedback training) to optimally engage targeted loci, as prior adult research, and compare such results with placebo conditions to test their power to change clinical and developmentally salient (i.e. self-processing and emotion regulation) outcomes.

A short version of the Emotion Self-Other Morph Query task before and after neurofeedback did not replicate lower mid-temporal limbic function (amygdala and hippocampus) in depressed vs. controls for happy self-other face recognition (Quevedo et al., 2018). This is likely due to smaller sample or/and shorter task duration (Present duration = 6.06 min, Prior duration = 10.54 min.). Additionally, earlier samples were significantly more depressed (Prior sample M_CDRS_ = 61.23, SD = 14.62 vs. Present sample: M_CDRS_ = 49.77, SD = 16.87). Different block orders would have impeded time and slope effects’ modeling in a small sample. Lack of counterbalanced orders might have impeded replication of prior results.

We sought to implement a novel neurofeedback protocol, and test depressed and controls’ self-processing during, before, and after the task. Therefore, decreased saliency network activity during self-face recognition after neurofeedback might be due to factors other than training. However, higher amygdala and hippocampus activity during neurofeedback was linked to higher amygdala and hippocampus during other-face recognition post versus pre- neurofeedback. Additionally, frontotemporal changes during self-faces recognition before vs. after neurofeedback suggest neuromodulation effects upon self-face recognition. A final limitation is the lack of baseline and transfer runs, which are recommended to determine neurofeedback success.

## 6 Conclusions

This is the first step for a future clinical trial comparing two targets: amygdala and hippocampus (upregulated in ROI analyses, mainly the hippocampus) and dorsal anterior cingulate cortex (engaged in whole-brain level analyses) activity. Our results suggest that longer amygdala and hippocampus neurofeedback duration or/and dorsal anterior cingulate cortex as a target of neurofeedback might be effective in adolescents, given our task’s brevity compared to prior adult neurofeedback tasks. Additionally, we ought to contrast dorsal anterior cingulate cortex with the hippocampus as loci of neurofeedback, because there could be developmental differences that make the dorsal anterior cingulate cortex a better locus in youth. Alternatively, future research could target the locus of neurofeedback in youth centered in the highest peak of dorsal anterior cingulate cortex activity in the current study versus a right amygdala or right hippocampus locus, given amygdala functional connectivity differences and dorsal anterior cingulate cortex, amygdala and hippocampus hypoactivity in depressed and suicide attempting youth during self-other face recognition (Alarcón et al., 2019; Quevedo et al., 2016;Quevedo et al., 2018).

## Funding

Funding awarded to KQ from the National Institute of Mental Health (NIMH; MH092601), Brain & Behavior Research Foundation (NARSAD Young Investigator Award, and the U of M Clinical and Translational Science Institute supported data collection, analysis and manuscript preparation. NZ’s work on this paper was supported by a National Research Service Award (NRSA) in Primary Prevention by National Institute on Drug Abuse through the Department of Psychology and the REACH Institute at Arizona State University (T32 DA039772). Funding sources were not involved in the design of this study, collection, analysis and interpretation of data or manuscript preparation.

## Declaration of Competing Interest

The authors declare that there is no conflict of interest regarding the publication of this paper.
