# Supplementary material for: Neurofeedback and neuroplasticity of visual self-processing in depressed and healthy adolescents: A preliminary study
Source: Dev Cogn Neurosci. 2019 Sep 11;40:100707. doi: 10.1016/j.dcn.2019.100707 (PMC6974905; doi:10.1016/j.dcn.2019.100707)
Supplement: Supplementary file 1 [file mmc1.docx]

**I. Online Steps to Warp the AMYHIPP ROI to each Individual Brain.**

**Generation of subject specific anatomic masks of the bilateral amygdala and hippocampus (AMYHIPP) region of interest (ROI).** The single band reference functional image from a pre-feedback multiband EPI series was used as the target functional reference for the coordinate system transformation since the neurofeedback would be generated from subsequent real-time multiband EPI within the same imaging session. The high-resolution structural image of the subject (MPRAGE) was also used as a structural anatomic reference for registration to the MNI reference. An AMYHIPP mask derived via the WFU_PickAtlas tool (Maldjian, Laurienti, Kraft, & Burdette, 2003) was transformed from MNI space into subject’s functional imaging space using SPM12 modules The 4-step process was as follows: 1. Alignment of the subject’s functional and structural images series. 2. Segmentation and spatial normalization of the structural image to the MNI coordinate space and output of spatial normalization parameters that perform inverse deformation between coordinate spaces. 3. Warping of the ROI from MNI space to subject space using the spatial normalization parameters yielded by step 2. 4. Registration of the warped ROI to match voxel-for-voxel the specific subject’s functional image space to allow for real-time masking. Masks were converted from 8bit to 16 bit for compatibility with MURFI. This last step concluded with overlapping of the ROI on both structural and functional images. **Supplemental Figure S. 1.**


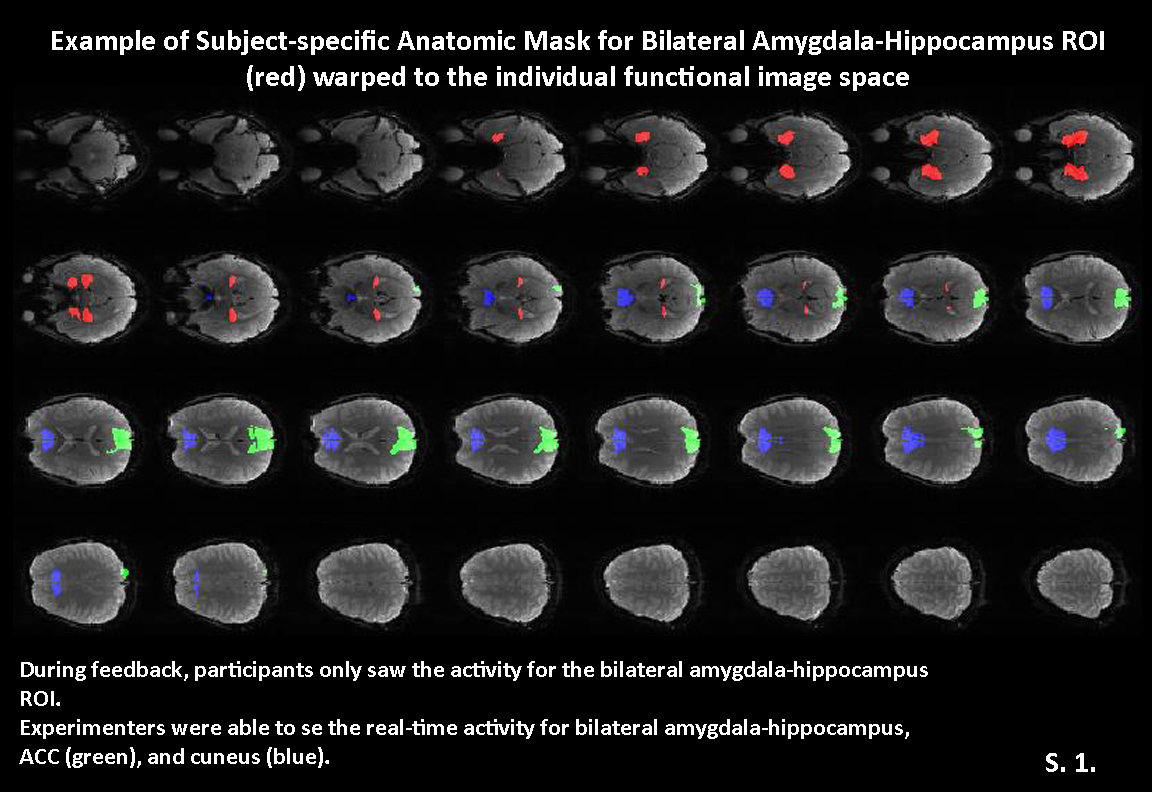


**II. Linear Mixed Model (LMM), mean amygdala and hippocampus (AMYHIPP) activity and Type III LMM results.**

**Generation of subject-specific AMYHIPP means.** To generate each subject-specific hippocampus and amygdala for each 8 blocks off line (4 Feedback and 4 Count backwards), the following expression in Matlab was used: *MeanActivityValue=nansum(Vcontrast_data(:).*Vmask_data(:))/nansum(Vmask_data(:)).*

**LMM.** To analyze subject-specific and mean AMYHIPP activity during the ESOM_NF task across the 8 blocks (See Supplemental Figure S.2.). The following LMM model (Equation 1 and 2) was used for ESOM_NF.

**Equation 1, Level 1 :** AMYHIPP _i t_ = β0i + β**x** i + δ

**Equation 2, Level 2 :**

β0 i t = γ00+δ0i,

β1 i t = γ01+δ1i,

β2 i t = γ02+δ2i**,**

β3 i t = γ03+δ3i,

β**x** i t = γ0**x**+δ**x**i

δ=error, t= time point, i = subject,

**ESOM_NF**: linear (γ01) = 0, 1 to 7, quadratic (γ02) = 0, 1, 4 to 49., cubic (γ03) = 0, 1, 8, to 343.

**X**= predictors e.g. Group, Gender, Slopes during ESOM_NF, Medication, IQ, etc.

**ESOM_NF Initial model with 22 predictors of interest**: Linear, Quadratic, Cubic, Task_Condition (FB or CB) Diagnostic_Group (Depressed or Control), Gender, Medication presence, IQ, Pre-happy-self_Amyhipp_, Pre-happy-other_Amyhipp_, Pre-neutral-self_Amyhipp_, Pre-neutral-other_Amyhipp_, Pre-sad-self_Amyhipp_, Pre-sad-other_Amyhipp_, Post-happy-self_Amyhipp_, Post-happy-other_Amyhipp_, Post-neutral-self_Amyhipp_, Post-neutral-other_Amyhipp_, Post-sad-self_Amyhipp_, Post-sad-other_Amyhipp_, RuminationChange, DepressionChange.

Upon observing the significance of separate emotion by self-face blocks, the Pre and Post AMYHIPP values were simplified to AllOtherPost – AllOtherPre and AllSelfPre – AllSelfPost. Models were compared via a χ ^2^ test of the -2LL fit difference. The more complex model after removing non-significant variables had 9 predictors, whereas the simpler model had 7 predictors and compared to the larger model the -2LL test yielded: -2LL_smaller model_ - 2LL_larger model_= 5.31, df =2 (NS), compared to the critical χ^2^ _(2)_ = 5.99. Therefore, the simpler model fitted the data better.

**Supplemental Figure S.2.** Mean Amygdala and Hippocampus activity within the Emotional Self-Other Morph Neurofeedback (ESOM_NF) task.


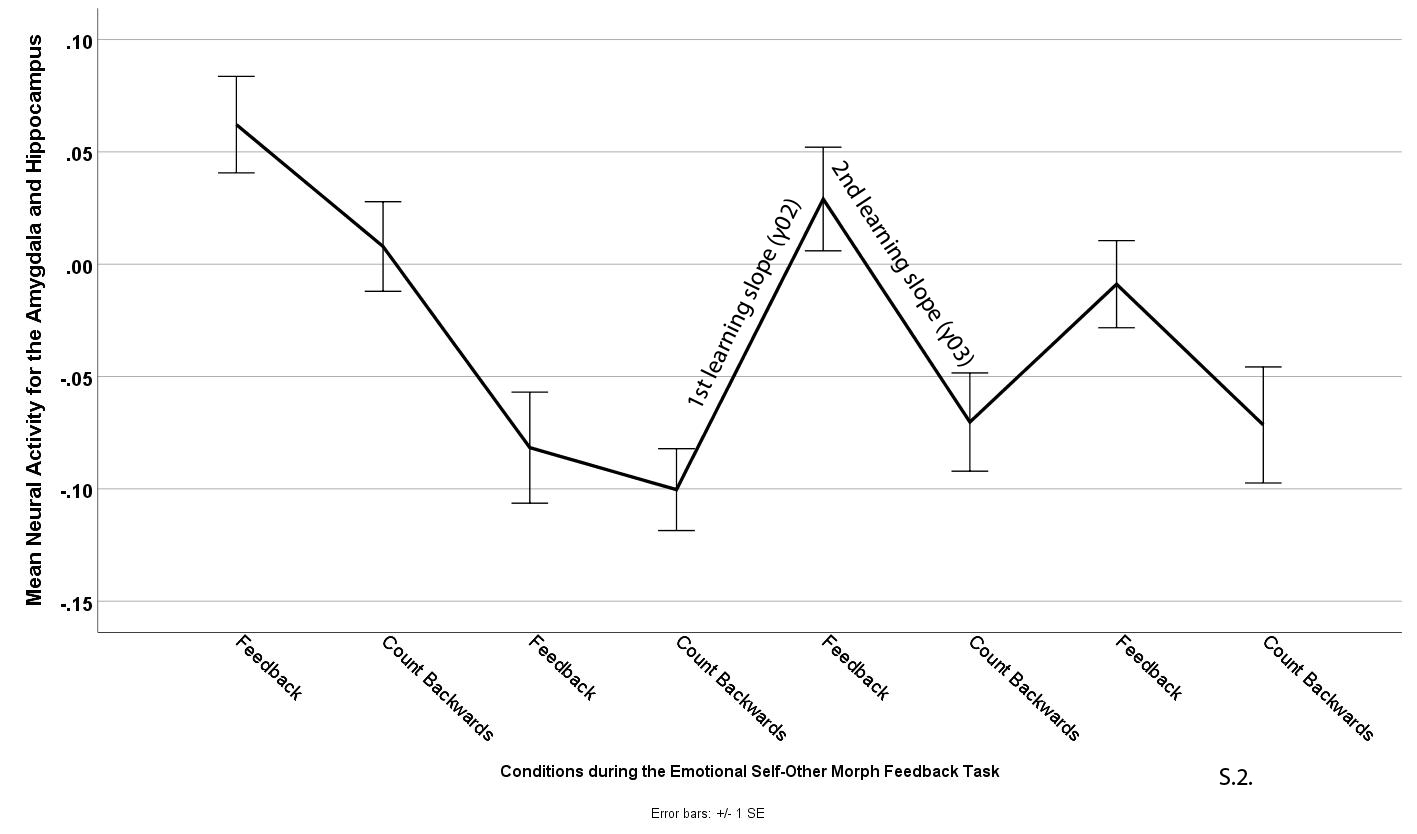


**III. Linear Mixed Model Analysis of Mean Amygdala and Hippocampus Activity Significant Predictors.**

| **Supplemental Table 1.**  **ESOM_NF Task: Type III tests for linear mixed model (LMM) Analysis of Mean AMYHIPP activity** | | | | |
| --- | --- | --- | --- | --- |
| Effect | Numerator df | Denominator df | *F* | *Sig. p* |
| Intercept | 1 | 324.93 | 10.45 | <.01 |
| Gender | 1 | 52.89 | 4.37 | <.05 |
| Feedback vs. Count-Backwards Condition | 1 | 370.75 | 5.10 | <.05 |
| Other-face Post – Other-face Pre AMYHIPP activity | 1 | 53.18 | 4.23 | <.05 |
| Linear Slope | 1 | 370.75 | 18.12 | <.01 |
| Quadratic: 1st Learning Slope (**γ02 _ESOM_NF_**) | 1 | 370.78 | 12.46 | <.01 |
| Cubic: 2^nd^ Learning Slope (**γ03 _ESOM_NF_**) | 1 | 370.75 | 9.39 | <.01 |
| Group * Quadratic (**γ02 _ESOM_NF_**) Interaction | 1 | 339.06 | 4.13 | *<*.05 |

**VI. Memory check and happiness before versus NF.** Ratings of happiness before and after neurofeedback (NF) and memory recall ratings were analyzed with a repeated measures ANOVA and a one-way ANOVA respectively. A 10-point scale rating measured successful recalling of happy memories during the ESOM_NF task, and happiness before and after NF. There were no significant differences, *F*(1,48)=0.397, p = 0.53, for ratings in successful recalling of happy memories between control (M=5.53) and depressed (M=5.18) groups. Additionally, analyses of ratings for happiness before and after ESOM_NF task shows that healthy control youth tended to have a higher rating in happiness overall at both times. However, the differences between group was not significant, *F*(1,50)=2.91, p=0.09 and the groups did not differ in their ratings before and after the ESOM_NF task *F*(1,50)=2.91, p=0.09.

**V. Whole-brain activity intensity maps.**


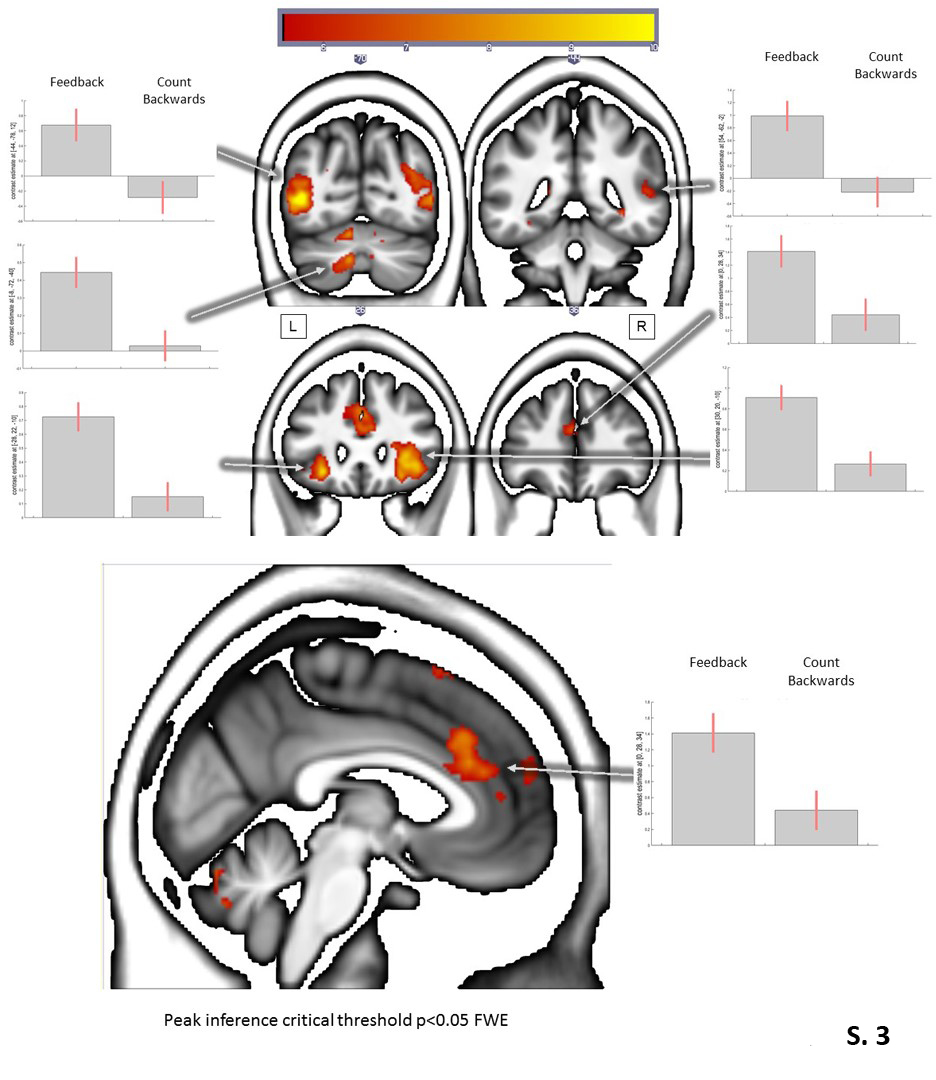
**Figure S.3 Whole brain activity intensity maps for the Emotion Self-Other Morph Neurofeedback Task.**


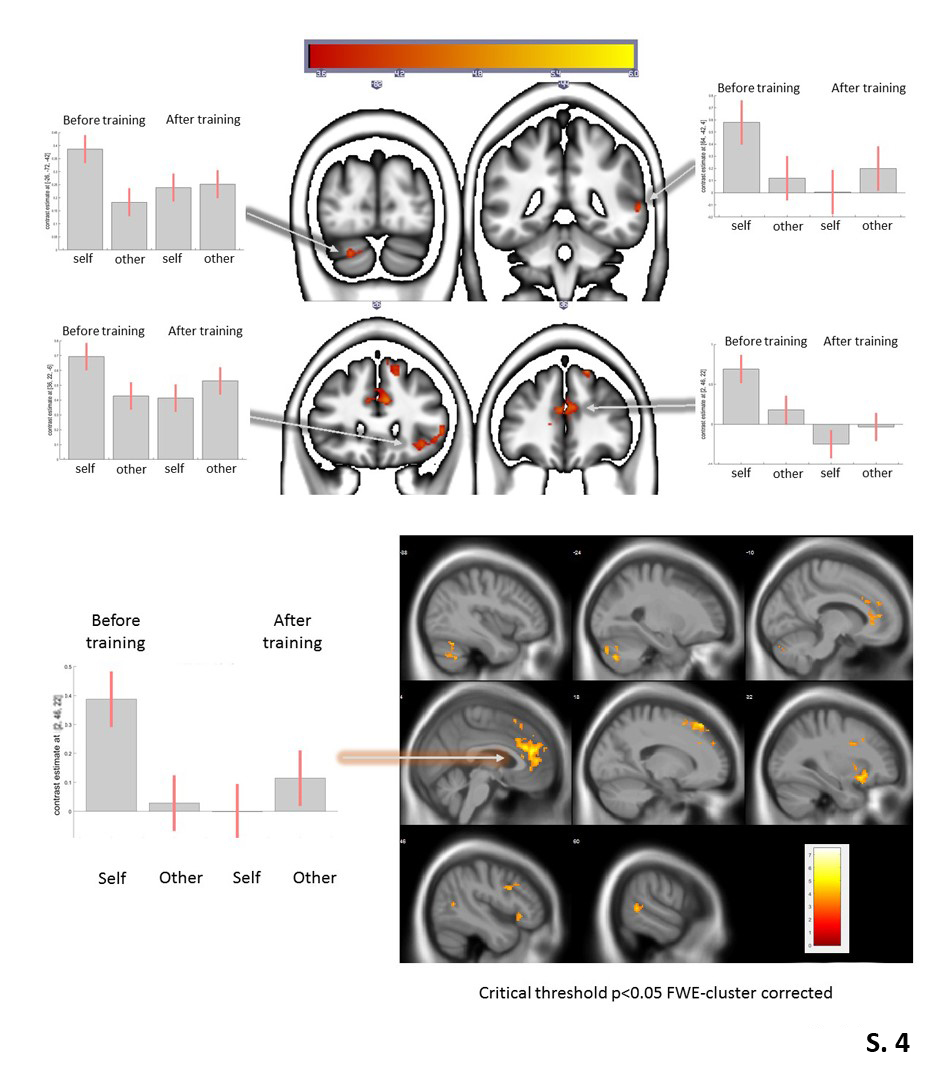
**Figure S.4** Whole brain activity intensity maps for the Emotional Self-Other Morph (ESOM) task that entailed self vs. other face recognition after (ESOM_Pre) versus before (ESOM_Post) neurofeedback training. Self-face recognition elicited less activity in a number of cortical areas after versus before a neurofeedback training task that included a neurofeedback condition cued by the self-face plus positive autobiographical memory recall.


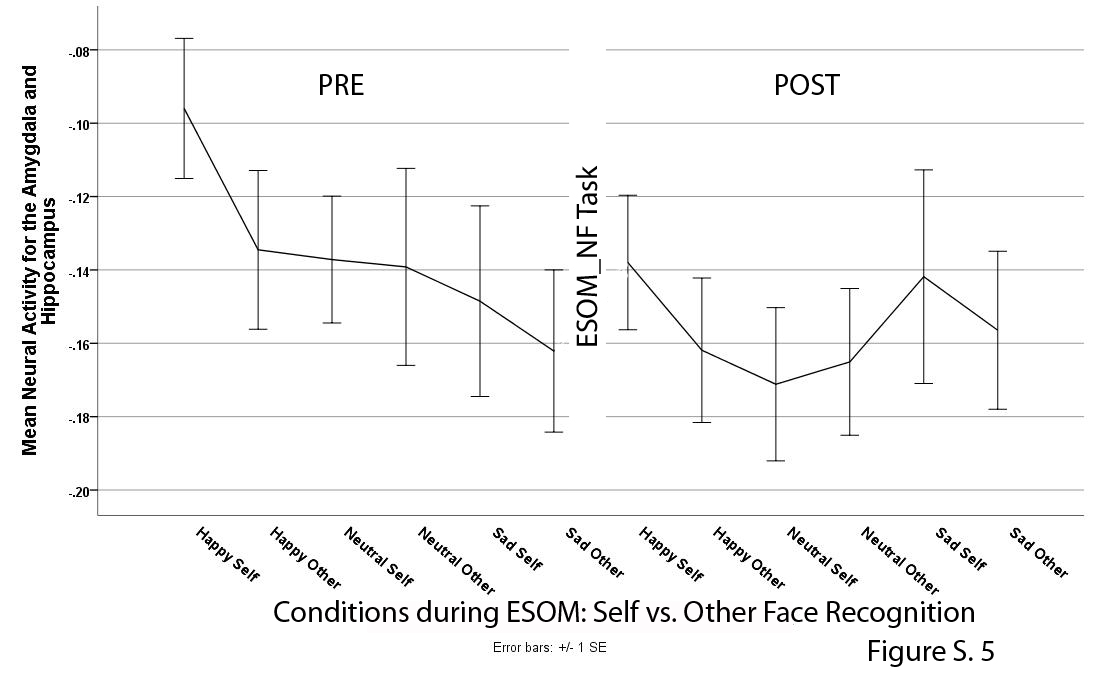


**Figure S.5** Mean AMYHIPP activity levels during self vs. other face recognition in the ESOM task pre and post the neurofeedback paradigm (Emotional Self-Other Morph Neurofeedback Task) also evidence significant higher initial values that decrease as the task continues.


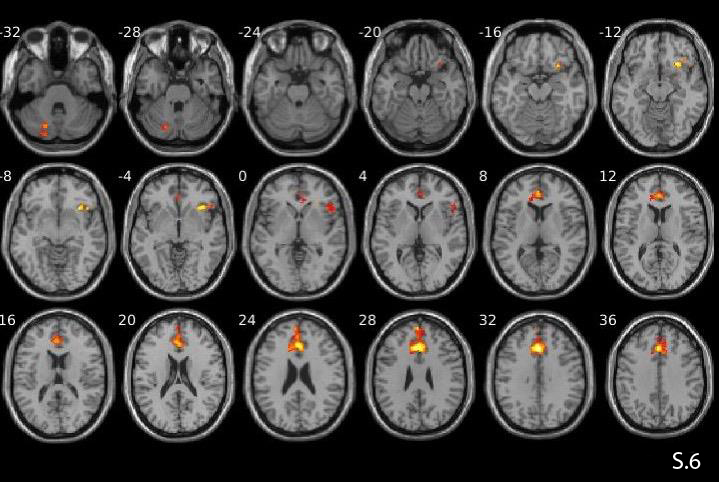


**Figure S.6** Clusters including the anterior cingulate cortex extending to dorsomedial prefrontal cortex and middle cingulate cortex as well as the insula extending to lateral orbitofrontal cortex and inferior frontal gyrus, and the cerebellum were commonly activated in the contrasts of feedback vs count-backwards during the ESOM_NF task and in the self vs other-face recognition during the ESOM_Post vs ESOM_Pre task.

References

Maldjian, J. A., Laurienti, P. J., Kraft, R. A., & Burdette, J. H. (2003). An automated method for neuroanatomic and cytoarchitectonic atlas-based interrogation of fMRI data sets. *Neuroimage, 19*(3), 1233-1239.
